# Comparison of Classifiers for Decoding Sensory and Cognitive Information from Prefrontal Neuronal Populations

**DOI:** 10.1371/journal.pone.0086314

**Published:** 2014-01-23

**Authors:** Elaine Astrand, Pierre Enel, Guilhem Ibos, Peter Ford Dominey, Pierre Baraduc, Suliann Ben Hamed

**Affiliations:** 1 Centre de Neuroscience Cognitive, UMR 5529 CNRS-Université Claude Bernard Lyon I, Bron, France; 2 Stem Cell and Brain Research Institute, INSERM U846-Université Claude Bernard Lyon I, Bron, France; Centre national de la recherche scientifique, France

## Abstract

Decoding neuronal information is important in neuroscience, both as a basic means to understand how neuronal activity is related to cerebral function and as a processing stage in driving neuroprosthetic effectors. Here, we compare the readout performance of six commonly used classifiers at decoding two different variables encoded by the spiking activity of the non-human primate frontal eye fields (FEF): the spatial position of a visual cue, and the instructed orientation of the animal's attention. While the first variable is exogenously driven by the environment, the second variable corresponds to the interpretation of the instruction conveyed by the cue; it is endogenously driven and corresponds to the output of internal cognitive operations performed on the visual attributes of the cue. These two variables were decoded using either a regularized optimal linear estimator in its explicit formulation, an optimal linear artificial neural network estimator, a non-linear artificial neural network estimator, a non-linear naïve Bayesian estimator, a non-linear Reservoir recurrent network classifier or a non-linear Support Vector Machine classifier. Our results suggest that endogenous information such as the orientation of attention can be decoded from the FEF with the same accuracy as exogenous visual information. All classifiers did not behave equally in the face of population size and heterogeneity, the available training and testing trials, the subject's behavior and the temporal structure of the variable of interest. In most situations, the regularized optimal linear estimator and the non-linear Support Vector Machine classifiers outperformed the other tested decoders.

## Introduction

Decoding neuronal information is an important analysis tool in neuroscience both as a means to understand how neural information is distributed and multiplexed over large populations [1], [2], [3], [4], [5], [6], [7], [8], [9], [10], [11], and as a means to drive neuroprosthetic effectors [12], [13], [14], [15]. In this framework, classifiers are used to define the most probable state of a given variable (the position of a stimulus in space, the direction of the intended motor plan etc.), given the observed instantaneous simultaneous activity of a neuronal population. Above-chance decoding accuracy indicates that the neuronal population contains reliable information about the variable of interest, whether its individual neurons also do or not.

In order to optimize their prediction, all classifiers define a decision boundary in the space of the variable of interest (2-D space for stimulus position or movement goal, n-class discrete space for stimulus or movement classification), using a training set of data, i.e. a set of neuronal population activities matched with the actual experimental condition that they correspond to. The accuracy of the decoders is then evaluated on a testing set of data, corresponding to neuronal population activities from an independent sample. Accuracy is calculated as the percentage of correct predictions provided by the classifier. The shape and properties of the decision boundary varies across classifiers. Linear classifiers will set hyperplane boundaries while non-linear classifiers will set complex non-planar boundaries. Flexible decision boundaries will maximize the separation of the training neuronal population response as a function of the decoded variable, including irrelevant idiosyncratic noise patterns specific of this training data. This over-fitting of the decision boundary will result in a poor generalization on new testing data (see [16], [17]). In contrast, a too simple decision boundary, such as a hyperplane, may often fail to account for a non-linear encoding of the variable of interest by the recorded neuronal population.

Most classifiers have been developed in the fields of statistics and machine learning. As a result, their mathematical properties are well understood. Early studies have formalized the use of major classifiers to the readout of continuous variables (such as position in space, orientation etc.) from neuronal population activities [18], [19]. However, in the face of real data, the sensitivity with which information is extracted from neuronal activity will depend on several factors. In particular, a given neuronal population may not encode with the same reliability and discrimination power all the variables it represents (e.g. a sensory information as compared to a cognitive information). As a result, classification sensitivity will depend both on the general response properties of the neuronal population being targeted and on the variable being decoded. The decoding sensitivity will also depend on the classifier being used as well as on the adequacy of the classifier with the experimental constraints. Several studies have used two or more decoders at reading out neuronal population activities (e.g. [20], [1], [2], [5]), without however pursuing a systematic comparison of their performance and how it is affected by the properties of the experimental data. In the following, we compare the readout performance of six commonly used classifiers operating on monkey frontal eye fields (FEF) spike signals, as a function of the size of the neuronal population, the number of training trials, and the balance in the data. The classifiers fall into three general decoder classes: probabilistic decoders, linear decoders and non-linear decoders. The classifiers we focus on are classifiers that have been used or proposed to decode neuronal population activities (non- exhaustive selection). These are a regularized optimal linear estimator, in its explicit formulation (*regularized OLE*, [12]) or in its linear artificial neural network approximation (ANN *OLE*, [1], [2]), a non-linear artificial neural network estimator (ANN *NLE*, [1], [2]), a non-linear naïve Bayesian estimator (*Bayesian*, [21]; please note that the naïve Bayesian estimation is formally equivalent to a Maximum likelihood classification) and a non-linear support vector machine classifier (*SVM*, [4]). A non-linear *Reservoir* recurrent network classifier (*Reservoir*, [22]) has also been tested because of its potential interest in decoding variables that have a specific organization in time. The general architecture and properties of these classifiers are described in the methods section.

We will compare how these decoders read out two distinct types of information available in FEF neuronal population responses. The first decoded variable corresponds to the position at which an initial stream of visual stimuli is presented. This information is exogenously driven by the environment (the presentation of the visual streams) and is robustly represented in the FEF [23], [24]. The second variable corresponds to the interpretation of the instruction held by the cue and the corresponding attention orientation signal. This information is endogenously driven in that it corresponds to the output of internal cognitive computations performed on lower level exogenous characteristics of the cue (here, position and color). Such endogenous attentional information is known to build up in the FEF [4], [25] and to influence lower visual areas, thanks both to feedback [26] and feedforward connections [27], [28].

In summary the present work pursues two objectives: 1) investigate whether endogenously driven neuronal information can be decoded with the same performance as exogenously driven neuronal information, and 2) identify the classifier that performs best at decoding neuronal information as a function of the experimental factors (neuronal population properties, subject's behavior and number of trials).

## Methods

### Ethical statement

All procedures were in compliance with the guidelines of European Community on animal care (European Community Council, Directive No. 86–609, November 24, 1986). All the protocols used in this experiment were approved by the animal care committee (Department of Veterinary Services, Health & Protection of Animals, permit number 69 029 0401) and the Biology Department of the University Claude Bernard Lyon 1. The animals' welfare and the steps taken to ameliorate suffering were in accordance with the recommendations of the Weatherall report, “The use of non-human primates in research”. The study involved two *Rhesus maccaca* (a male, 10 kg, age 7 and a female, 7 kg, age 6), a standard in electrophysiological studies. The animals were housed in twin cages (2 m^2^ by 2 m height in total). The twin cages could be separated in two individual cages or on the opposite, connected to form a unique housing for a pair of monkeys thus offering the monkeys a socially enriched environment. This last configuration was the norm. Twin cages communicated with a larger play cage (4×1.5×2 m^3^) to which the monkeys were granted access on days on which they were not involved in experiments. Light was switched on and off at fixed hours (on: 7.30 a.m and off: 8 p.m), all year round. Monkeys had free access to food pellets. They were also given fresh fruits and nuts. All cages were enriched with mirrors, hanging ropes, water pools, balls and foraging baskets. No procedure that might cause discomfort or pain was undertaken without adequate analgesia or anesthesia. In particular, each monkey underwent a single surgical session under gas anesthesia (Vet-Flurane à 0.5–2%) during which a craniotomy was made over the left (resp. right) prefrontal cortex for monkey Z (resp. M) and peek recording chambers were implanted to allow access to the FEF with microelectrodes. Post-surgery pain was controlled with a morphine pain-killer (Tamgesic, 0.01 mg/kg i.m.) and a full antibiotic coverage was provided (long action Tamgesic 100, one injection during the surgery and one 5 days later, 0.1 mg/kg, i.m.). The general health status of the animals was monitored every day by competent and authorized personal. In agreement with the 3R ‘reduction’ recommendation, the two animals involved in the present study were enrolled later in another experiment.

### Description of the neurophysiological database

#### Behavioral task

**Figure 1:**
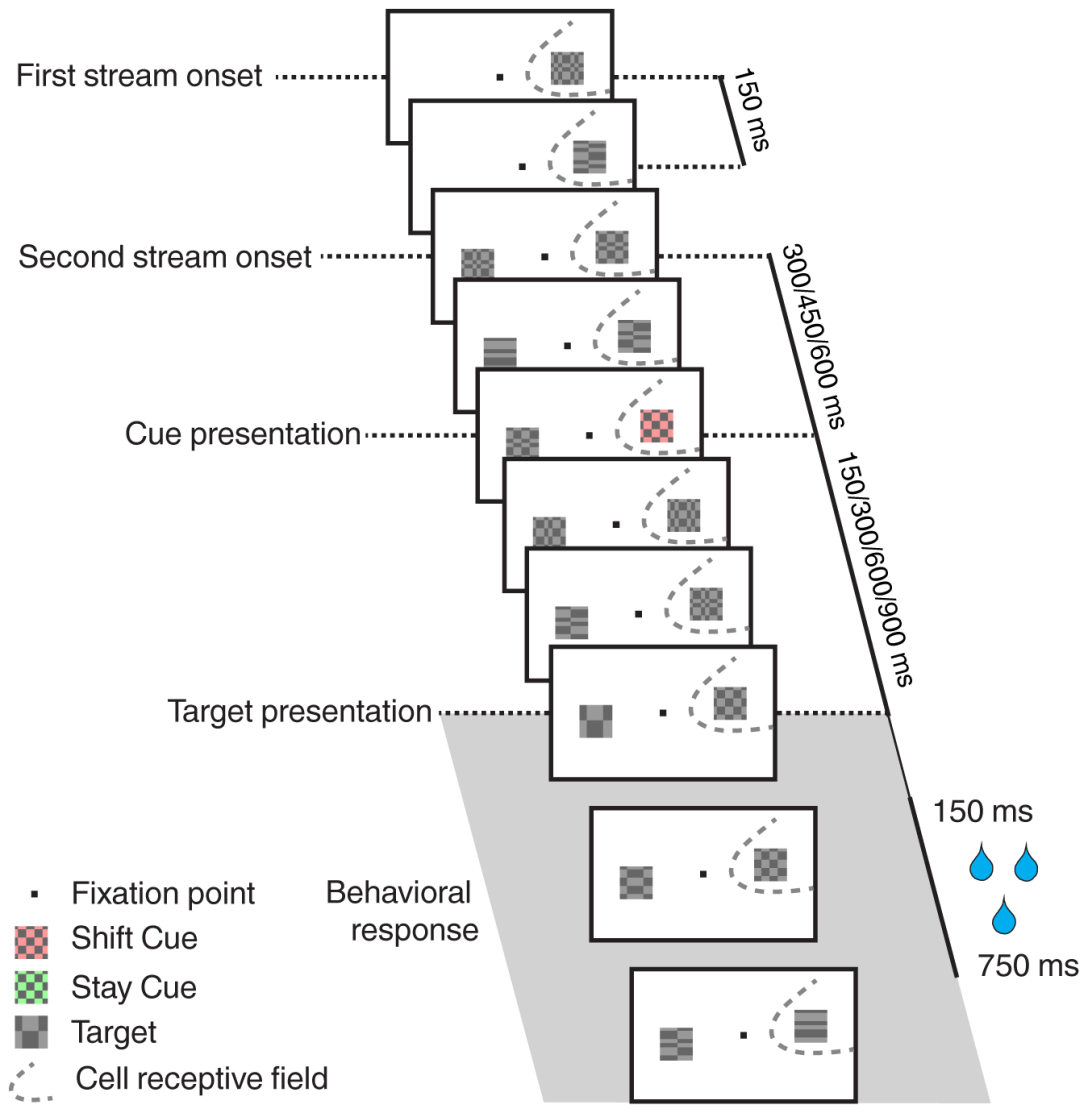
Task description. The experimental procedure is a cued-target detection based on a dual rapid serial visual presentation (RSVP) paradigm. The monkey is required to maintain its gaze on the central fixation point all throughout the trial. A first stream of stimuli, that is a succession of visual stimuli every 150 ms, is presented either within (as here) or opposite the fixation point from the cell's receptive field. Three hundred milliseconds later, a second stream appears opposite the first stream from the fixation point. Three hundred, 450 or 600 ms (here, 300 ms) following the second stream onset, a cue is presented within the first stream. This cue can be a green stay cue indicating to the monkey that the target has a high probability to appear within this very same stream or a red shift cue (as here), indicating that the target has a high probability to appear within the opposite stream. On 80% of the trials, the target is presented 150, 300, 600 or 900 ms from cue onset. On 80% of these target trials (64% of all trials), the target location is correctly predicted by the cue (valid target, as here). On 20% of these target trials (16% of all trials), the target location is incorrectly predicted by the cue (invalid target). On the remaining 20% of trials, no target is presented (catch trials), so as to discourage false alarms. The target is composed of just one horizontal and one vertical spatial cycle, while distractor items are composed of up to 6 horizontal and vertical spatial cycles. The monkey gets rewarded for responding by a bar release, between 150 and 750 ms following target presentation, and for holding on to the bar when no target is presented.

The data analyzed in the present work were collected while monkeys performed a cued target detection task based on a rapid serial visual presentation (figure 1, see also [25], [29]). It allowed to dissociate in time the processes related to the orientation of attention from those related to target detection [30]. In particular, the cue was a non-spatial abstract cue that informed the monkey in which hemifield it should direct its attention. Briefly, the monkey had to fixate a central point on the screen throughout each trial. Two streams of visual objects were presented, one in the visual receptive field of the neuron being recorded and the other in the contralateral hemifield. One of the streams included a cue which instructed with a certain probability the position of the target. The cue could be green (resp. red), predicting that the target would appear in the same (resp. other) stream. In the following, the green cue will be called a *Stay* cue and red cue a *Shift* cue. The monkey had to combine the information related to the physical attributes of the cue (its location and its color) to find out where the target was likely to appear. The monkey had to release a lever to report the presence of the target. The target appeared on 80% of the trials. The remaining 20% no target trials were catch trials that served to discourage the monkeys from making false alarms. In target trials, the target appeared either 150 ms, 300 ms, 600 ms or 900 ms following the cue. In 80% of these trials (64% of all trials), the target appeared in the instructed stream (valid trials). In the remaining 20% target trials (16% of all trials), it appeared in the opposite stream (invalid trials). The monkey was rewarded for releasing the lever 150 to 750 ms following target onset on valid and invalid trials and holding it on catch trials. Invalid trials were used to check that the monkey used the predictive information provided by the cue in order to guide its behavior. Sessions in which this was not the case were discarded from the analysis.

#### Cell population

The spiking activity of 131 frontal eye field (FEF) neurons was recorded from two macaque monkeys. All procedures were approved by the local animal care committee in compliance with the guidelines of the European Community on Animal Care (cf. [25] for details). These cells were subjected to individual statistical analysis. Amongst them, a subset of neurons (n = 21) reliably encoded cue instruction while apparently providing no information about cue location or cue color (see [25] for details). These cells thus encoded the final position of attention, discriminating between cues instructing attention towards the receptive field (contralateral *Stay* cues and ipsilateral *Shift* cues) and cues instructing attention away from the receptive field (ipsilateral *Stay* cues and contralateral *Shift* cues). In the following, we will be comparing the performance of several classifiers at decoding the position of the initial visual stream of stimuli (exogenously driven visual information) to their performance at decoding the final position of attention instructed by the cue (endogenously driven cognitive information). The exogenous information was decoded using the entire FEF neuronal population while the endogenous information was decoded using either the entire population or the subset of cue instruction cells. This allows us to make two comparisons. Firstly, we can compare the decoding performance for exogenous versus endogenous information using the entire FEF neuronal population, secondly we can compare the decoding performance of endogenous information between the entire FEF neuronal population and the subset of cells that we previously identified as significantly modulated by the variable of interest.

### Decoding procedure

#### Data pre-processing

For each cell and each trial, the spiking data was smoothed by averaging the spiking activity over 100 ms sliding windows (temporal resolution of 1 ms). This window width corresponds to a trade-off between performance and decoding speed, as narrower filtering windows result in a lower performance while wider filtering windows increase the delay of real-time decoding [29]. The 131 cells were combined to form a single neuronal population. To decode the first flow position, both correct and error trials were used, because the cells' response to this exogenous event does not depend on the monkey's engagement in the task. As a result, an average of 295 trials (s.d. = 107) was available per cell. In contrast, only correct trials were used to decode the position of attention, as error trials can arise from an improper orientation of attention. In addition, unless otherwise stated, trials in which the target appeared 150 ms after the cue were excluded from the data set to avoid a confound between cue- and target-encoding. As a result, fewer trials were available (mean = 112 trials, s.d. = 33). For each cell, 60 trials were randomly selected per condition (First flow on the left, First flow on the right, Attention instructed to the left and Attention instructed to the right). For most cells, these trials corresponded to a random subset of all the available trials per condition. For a minority of cells, some trials were randomly duplicated to achieve the requirement of 60 trials per condition. Since this can potentially induce an artificial inflation of decoding performance, we conducted random permutations following the exact same procedure as described in the data pre-processing section, in order to define the actual chance level; decoding performance was systematically compared to this chance level. Single trial responses were randomly combined across the entire neuronal population in order to create 60 virtual population responses to each event of interest. This procedure, defining a seed population activity, was repeated 20 times, thus defining 20 different population activity seeds (out of more than 131 to the power of 60 possible population activities, thus limiting the potential inflation induced by the duplication of some trials). Note that these population responses are free of the correlations that would be found in simultaneous recordings.

#### General cross-validation procedure

Visual and attention-related signals do not have the same temporal dynamics and their mean response peaks at different latencies from event onset. For both variables, the decoding was performed around this peak response. As a result, when decoding the position of the initial visual stream, we trained the classifiers on the smoothed activity observed at 125 ms following visual stream onset (i.e. on the 100 bin centered at 125 ms). When decoding the instructed position of attention, we trained the classifiers on the smoothed activity observed at 245 ms following visual stream onset (i.e. on the 100 bin centered at 245 ms). These timings correspond to the timing of the peak neuronal response to each specific event as estimated in Ibos et al. [25]. Due to a more complicated architecture, the reservoir was trained using data from a time-window of 75 ms around these training references.

We trained the classifiers on 70% of the data (84 trials) and tested them on the remaining 30% of the data (remaining 36 trials) so that the testing is performed on a naïve set of trials, never experienced by the classifier. During *training*, the decoders were simultaneously presented with single-trial population activities (corresponding to the observed inputs) and the state of the decoded variable (corresponding to the associated outputs: Visual stream on the left or on the right or Attention instructed to the left or to the right). During *testing*, the decoders were presented with the successive test sets centered on a window of 100 ms around the time at which training was performed (i.e. one test set every 1 ms in this window) and produced their guess for the state of the decoded variable. The readout performance on each decoding run is then calculated by averaging the performance produced by the 100 successive testing sets (1 ms resolution) and corresponds to the percentage of trials on which the classifier provided the correct guess for the state of the decoded variable. This procedure was chosen to ensure that the final readout performance reflects a robust pattern of activity. This *training*/*testing* procedure was repeated for each data seed (i.e. 20 times in all, cf. data pre-processing section) to yield an average readout performance, using the exact same randomly constructed *training*/*testing* datasets for all decoders. Testing the decoders on a set of predefined seeds allows to discuss their readout performance independently of data variability.

#### Random permutation tests

Randomized permutation tests were performed for each classifier and for each analysis using the exact same procedure as above, after assigning, for each cell, randomized condition labels to each trial (using a random sampling with replacement procedure). This procedure, repeated 50 times, for each of the 20 data seeds, yielded the distribution of chance performance of each classifier. This distribution was thus constructed with 1000 data points. The readout performance of a given classifier was considered as significant when it fell in the 5% upper tail of its corresponding chance performance distribution (non-parametric random permutation test, p<0.05).

### Classifiers

#### Optimal Linear Estimator (OLE)

**Figure 2:**
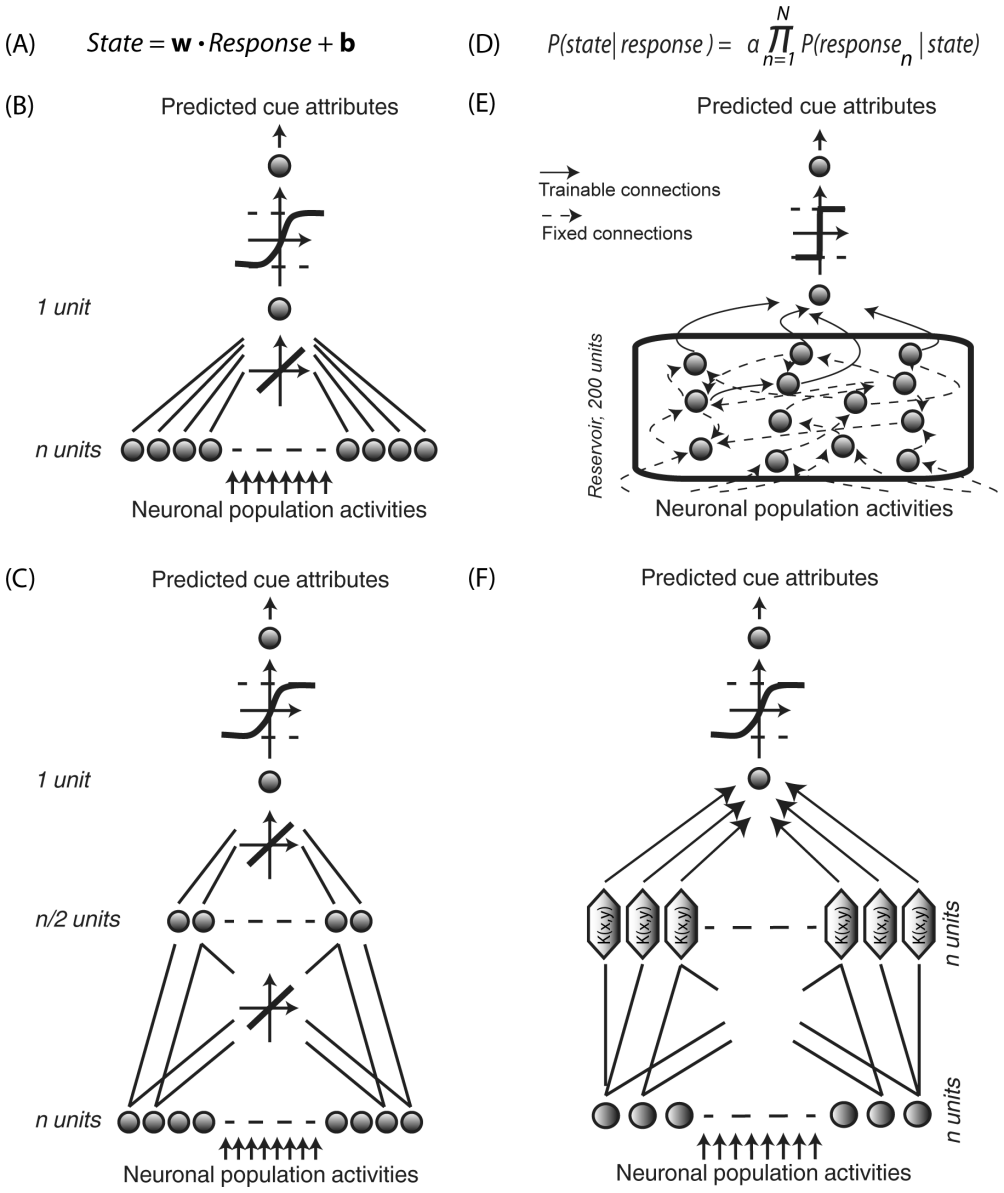
Decoders. (A) Regularized OLE, the training step is a simple regularized linear regression. (B) Optimal Linear Estimator (ANN OLE), implemented as a one-layer feedforward artificial neural network. The input layer has one unit per FEF cell and receives instantaneous population neuronal activities. The output layer contains 1 unit. Training involves optimizing the weights using a Levenberg-Marquardt backpropagation algorithm and a hyperbolic tangent transfer function. (C) Non-Linear Estimator (ANN NLE), implemented as a 2-layer feedforward artificial neural network. The network architecture only differs from the OLE by an additional hidden layer with n/2 units, n being equal to the number input units. (D) Bayesian decoder, applying Bayes' theorem to calculate the posterior probability that state i is being experienced given the observation of response r. (E) Reservoir decoding. The decoder has one input unit per FEF cell and one output unit. Fixed connections are indicated by dotted arrows and dynamical connections are indicated by full arrows. The reservoir contains 200 units. The recurrent connections between them are defined by the training inputs. A simple linear readout is then trained to map the reservoir state onto the desired output. (F) Support Vector Machine (SVM), the LIBSVM library (Chih-Chung Chang and Chih-Jen Lin, 2011) was used (Gaussian radial basis function kernel so as to map the training data into a higher dimensional feature space). The transformed data is then classified with a linear regressor and training is performed with a 5-fold cross-validation. For all decoders, the sign of the output corresponds to the two possible states of the variable being decoded.

**Figure 3:**
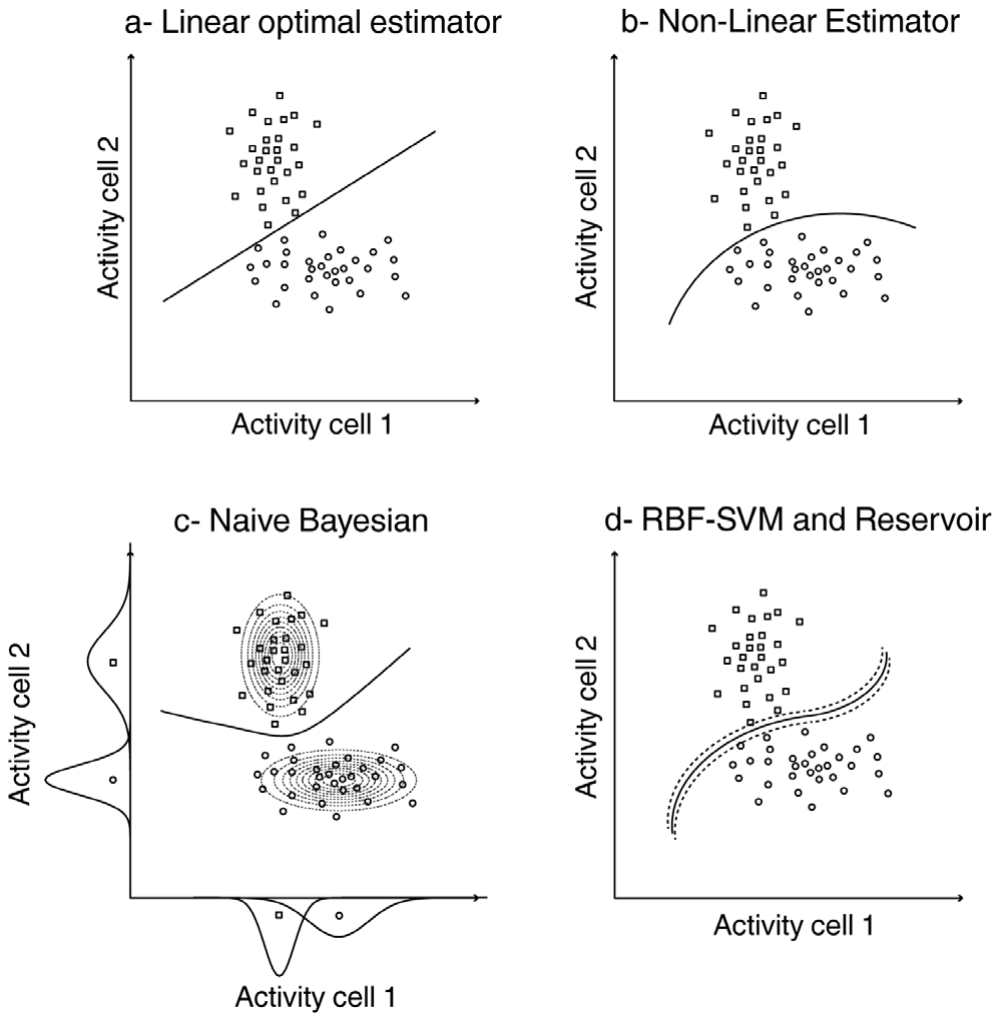
Decision boundaries for the different classifiers. Each plot represents the activity of a hypothetical cell 1 as a function of the activity of hypothetical cell 2, on successive trials, in response to a stimulus 1 (circles) or 2 (squares). a) Optimal linear estimator; b) non-linear estimator; c) naive Bayesian. The dotted ellipsoids (Bayesian) correspond to the probability-density fitted Gaussian distributions of the cells' activities for each stimulus; d) SVM with Gaussian kernel (RBF) and Reservoir. In the case of SVM, the dotted line corresponds to the margin around the decision boundary.

The linear regression (figure 2a–b) minimizes the mean square error for the following equation **C** =  W*R, where **R** is an n by t matrix of **R**_ij_, n being the number of cells in the neuronal population of interest, t the number of available trials and **R**_ij_ the neuronal response of cell i in the population, on trial j; **C** is a 1 by t vector, the sign of the elements of which describes the two possible classes taken by the binary variable of interest and **W** is a 1 by n vector corresponding to the synaptic weights that adjust the contribution of each cell to the final readout. This procedure defines a linear boundary between data points sampled from two independent distributions (figure 3a). As a result, such an estimator is optimal provided the neuronal output of the population activity is a linear sum of the inputs. This assumption appears to be a general property of neuronal populations (see [19], [31], [32], [1], [2], who suggest that neurons could form a set of basis functions encoding real-world variables). Such a linear decoding can be achieved in two ways:

Regularized Explicit function (R. OLE). The first approach is to inverse the above equation as **W** =  **C** *R ^†^, noting **R** ^†^ the Moore-Penrose pseudo-inverse of **R**. **R** ^†^ was determined on a subset of the data (Figure 2a, A_train_ =  training dataset) and the resultant **W** matrix was applied to solve **C**  =  **R** * **W** on the rest of the data (A_test_ = testing dataset). As the Moore-Penrose pseudo-inverse leads to overfitting, we used a Tikhonov-regularized version of it: this solution minimizes the compound cost norm(W*R – C)+ λ*norm(**W**), where the last term is a regularization term added to the original minimization problem [33]. The scaling factor λ was chosen to allow for a good compromise between learning and generalization. Its precise value was optimized for each analysis as this value depended on the population size and number of training trials (see [34] for the λ optimization procedure).

Artificial Neural Network (ANN OLE). To estimate the penalty (or benefits) of training artificial neural networks, we compared the formal *OLE* solution described above with the performance of a one-layer feed-forward network with as many units in the input layer as in the FEF cell population of interest, one unit in the output layer reflecting the class of the binary variable of interest and a hyperbolic tangent transfer function (see Figure 2b, [1], [2]). Training was performed using a quasi-Newton back-propagation that defines the weight vector **W** which minimizes the square distance between the estimate of the state of the variable of interest and its actual value. To prevent overfitting, a regularization procedure was used. This procedure modifies the initially chosen network performance function (the mean of sum of squares of the network errors) by adding an additional regularization term. The regularization term consists of a weighted mean of the sum of squares of the network weights and biases. As a result, the modified performance function *msereg* becomes: msereg  =  λ*mse + (1−λ)*msw, where *mse* is the mean square error and *msw* is the mean square weight. The factor λ sets the performance ratio between the mean square error and the mean square weight. Here, equal weight was given to both the mean square error and the mean square weights (λ = 0.5) as this value yielded the highest decoding performances. The sign of the classifier output described the possible states of the variable of interest (−1 and 1).

#### Non Linear Artificial Neural Network Estimator (ANN NLE)

The *OLE* described above cannot, by definition, capture non-linear processes, which might be at play in prefrontal cortical regions and/or during cognitive endogenous processes. We thus decided to implement a non-linear estimator. If the *ANN NLE* outperformed the *ANN OLE*, this would support the presence of non-linear neuronal information processes. The *ANN NLE* is implemented similarly to the *ANN OLE* above, except that a second layer is added to the network architecture in order to capture potential hidden non-linearities in the neuronal population response. This additional hidden layer has half as many units as the input layer (Figure 2c). Such a two-layer network architecture draws a non-linear boundary between data points sampled from two independent distributions (figure 3b).

#### Bayesian classifier

We used a Gaussian naïve Bayes classifier [35], [36] which directly applies Bayes' theorem (Figure 2d) to calculate the conditional probability that the population response, ***R*** is of class *C_k_*: P(*C_k_*|***R***). Cells are “naïvely” assumed statistically independent. Bayes' theorem can be written for cell *n* as follows: 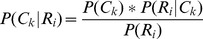
(eq. 1)


P(*R_i_*) can be ignored, since it is constant and independent of C_k_. P(C_k_) is also constant across the different classes by design (the two classes are equi-probable). As a result 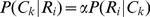
(eq. 2)where α is constant across the different classes C_k_. If the components *R_i_* of **R** are independent,
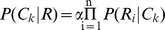
and the Bayesian classifier is optimal, in the sense that it intrinsically minimizes the misclassification rate. Indeed, misclassification is minimized if and only if the response **R** is assigned to the class C_k_ for which the P(C_k_|**R**) is maximum [37]. As a result, the Bayesian decoding procedure amounts to using f_k_(**R**)  =  P(**R**|C_k_) as discriminant function. We estimated the conditional probability density of the neuronal response R*_i_* of a given neuron P(R*_i_*|C_k_), given a stimulus class C_k_, as a Gaussian distribution, taking as parameters the mean and the standard deviation of the neuron's response across trials. The resulting Bayesian classifier draws a quadratic non-linear boundary between data points and takes into account the variance structure of the input distributions (figure 3c, distinct-variance Gaussian Bayesian model). This is equivalent to a discrete maximum likelihood method in that it calculates, for each trial, the probability of each class and chooses the class that presents the highest probability.

#### Reservoir Computing

We used a specific class of recurrent neural networks derived from *Reservoir computing*. In such a design, the dynamics of the neurons of the reservoir map the input onto a higher dimensional space, thus unveiling potential hidden contingencies. The simple readout process is then trained to map the overall state of the neuronal reservoir onto the desired output [38], [39]. Because of the higher dimensionality mapping achieved by the reservoir, such a recurrent neuronal network is expected to yield a better read out performance than a simple direct linear mapping (OLE) between the input and the desired output. In particular, it allows to segregate the data points sampled from two independent distributions thanks to a non-linear boundary that minimizes the mean square error in the higher dimensionality space the input data is projected on. Specifically, we used a recurrent neural network (RNN, figure 2e) with fixed connections and a readout layer that reads the activity of all neurons in the RNN [40]. All parameters specific to the reservoir were set with a grid-search procedure prior to the decoding experiments in order to optimize the decoding performance. This procedure consisted in testing the decoding performance of the reservoir over a large set of parameters and selecting those parameters that maximize correct classification. Due to heavy and time costly computations, these parameters (number of nodes, transfer function, scaling factor, input sparseness, reservoir sparseness, spectral radius, time constant and regularization parameter) were optimized only for full population- and trial-sizes. For all analyses, unless otherwise stated, the nonlinear optimal reservoir contained 500 analog nodes without transfer function. The fixed connections between the input units and the reservoir were randomly generated from a uniform distribution between 0 and 1 and scaled with a factor of 10^−1.2^ in order to balance how strongly the reservoir is driven by the input data. This optimal reservoir had no interconnections between its nodes. The nodes were initially set as leaky integrator, but optimization of their time constant revealed that the network performs better without leaky integration. As a result, such a reservoir is equivalent to a completely non-dynamic neural network using independent non-linear transformations to calculate the decoding performance. A Tikhonov regularization procedure was chosen in order to avoid overfitting. The readout layer performs an explicit linear regression between the activity of the neurons within the RNN and the desired output.

#### Reservoir with memory

Recurrent networks like the reservoir have been used to process temporal information such as time series. Here, we wanted to test whether the reservoir could extract temporal information embedded in the data and provide a stationary decoding performance that memorizes the decoded event for a longer period of time. To do this, new parameters were set in a grid-search manner (as described above) in order to optimize the decoding performance for a training window of 70 ms to 500 ms after cue onset. The non-linear dynamic reservoir contained 500 analog nodes with a hyperbolic tangent transfer function. The fixed connections between the input units and the reservoir were randomly generated from a uniform distribution between 0 and 1 and the scaling factor was set to 10^−3.8^. There were no interconnections between the nodes within the reservoir and the time constant was set to 55. These parameters created a non- dynamic reservoir that, because of the high time constant, uses previous time-steps to extract information. The readout layer performed an explicit linear regression between the activity of the neurons within the RNN and the desired output.

#### Support vector machine (SVM)

The basic SVM can be considered as a non-probabilistic binary linear classifier that maps the inputs in space so as to maximize the separation between the inputs of the two classes ([41], figure 2f). The input data is nonlinearly mapped to a higher-dimensional feature space and then separated by a maximum margin hyperplane. Generally, this maximum margin hyperplane corresponds to a non-linear decision boundary in the input space, defined by the following equation (Eq3)
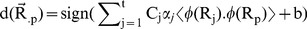
(eq. 3)where 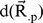
is the decision on the test neuronal population response 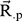; t is the total number of training trials; the class labels Cj ∈ {−1,+1} and represent the states of the binary output variable during training; α_j_ represents a set of t constants that define the SVM optimal solution for the training set; the input data vector 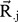
represents the population neuronal response on trial j. The decision boundary is fully defined by a subset of training samples, the so-called support vectors, but is never explicitly calculated. Mercer's theorem states that for each continuous positive definite function, K(**x**, **y**), there exists a mapping **Φ** such that K(**x**, **y**) equals the dot-product, <**Φ**(**x**),**Φ**(**y**)> for all **x**, **y** ∈ R^n^. Mercer's theorem allows to learn the relationship between **x** and **y** in the feature space without an explicit estimation of the mapping function **Φ**, by simply using a kernel function; this makes the support vector machine efficient for operating in a high-dimensional feature space [42], [43]. The architecture of the SVM decoder we use here is presented on figure 2f (LIBSVM library, Gaussian kernel implantation, [44], http://www.csie.ntu.edu.tw/~cjlin/libsvm). Note that we used a SVM design with a Gaussian kernel, K(x,y)  =  exp(−γ||x−y||^2^). Overall, because the input data is projected onto a higher-dimensional feature space, SVM allows segregating the data points sampled from two independent distributions thanks to a non-linear boundary (figure 3d). A grid search procedure (calculating decoding performance over a range of cost and gamma SVM parameters) was performed, for each set of train data, prior to the decoding procedure, in order to find the SVM parameters that maximize decoding performance. This was done using a 5-fold cross-validation procedure so as to minimize over fitting. Specifically, each training set was randomly divided into 5 parts. One part was retained for testing the model while the other 4 parts were used for the training of the grid search procedure. This procedure was repeated 5 times so that each part is used exactly once to evaluate the selected parameters.

## Results

Though the mathematical properties of the classifiers considered in the present work are well described, how they behave and how they differ when applied to real neuronal population activities has not been investigated this far. In particular, no study has directly questioned how their performance is affected by actual biological noise in the data, and how it differs between sensory and cognitive signals. In the following, we examine the performance of different classifiers and their dependency on several parameters that often turn out to be crucial in the context of single cell recording experiments. We first compare the decoders' performance as a function of the variable being decoded (visual/exogenous versus attentional/endogenous). We then evaluate the dependency of each decoder on the number of available training trials and the number of available cells. Last, we quantify the impact of unbalanced training samples, i.e. samples with unequal number of trials for each decoded class.

### Who's best? Comparing readout performance across classifiers

**Figure 4:**
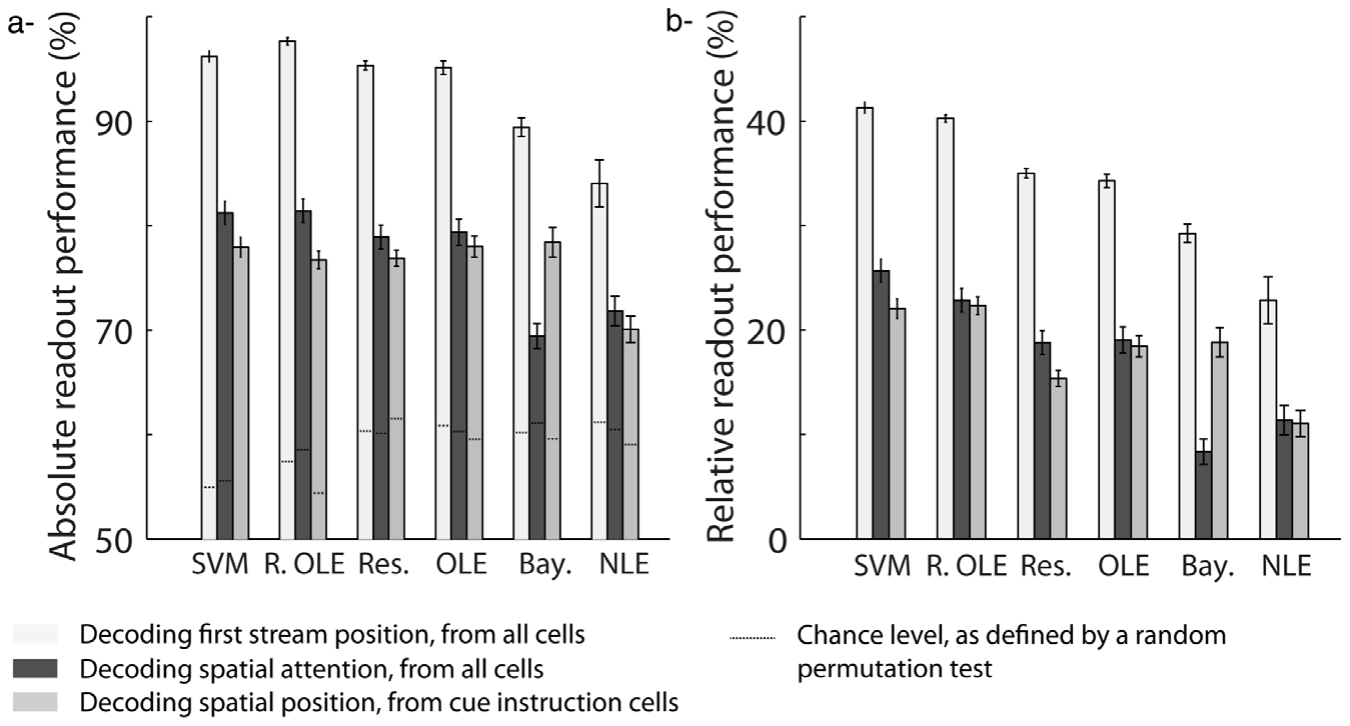
Comparison of mean performance at reading out first stream position and spatial attention across classifiers. A) Absolute readout performance. The dashed lines indicate the chance level for each condition, as estimated by a random permutation test (p<0.05). B) Readout performance, relative to chance level. The flow position is decoded using all cells in the population (light gray). Spatial attention is decoded using all cells in population (dark gray) or using only cells with significant individual attention-related responses (intermediate gray). The mean readout performance and the associated standard error around this mean are calculated over 20 decoding runs. SVM  =  support vector machine, Res.  =  reservoir, R. OLE  =  regularized OLE, Bay.  =  Bayesian, NLE  =  ANN non-linear estimator, OLE  =  ANN optimal linear estimator.

A straightforward measure of how well a decoder extracts information from population neuronal activities is its readout performance, i.e. its correct classification rate. We thus compared the average performance of each classifier (*SVM, Reservoir, regularized OLE, Bayesian, ANN OLE and ANN NLE*) over 20 successive decoding runs (each performed on a distinct data set, cf. data seeds in the methods section) when decoding either the position of the first visual flow (figure 4a, light gray bars), or the instructed position of attention (figure 4a, dark gray bars) from the whole FEF population (n = 131). The different classifiers did not perform equally well and this, irrespectively of whether the position of the first visual flow or the instructed position of attention was being decoded (2-way repeated measure ANOVA, Variable x Classifier, Classifier main factor, p<0.001, figure 4a). A Bonferroni post-hoc analysis indicated that the *SVM*, the *regularized OLE,* the *Reservoir* and *the ANN OLE* significantly outperformed *the Bayesian and the ANN NLE* (p<0.001) both when decoding position of the first visual flow (p<0.01) and the instructed position of attention (p<0.001).

However the 95% confidence interval (as estimated by a non-parametric random permutation test, p<0.05, cf. methods) that served as a decision boundary for significantly above chance performance varied from one decoder to the other. We calculated, for each classifier, its performance relative to this 95% confidence upper limit (figure 4b). As was the case for the absolute readout performance, the relative readout performance also varied across classifiers (2-way repeated measure ANOVA, Variable x Classifier, Classifier main effect p<0.001, figure 4b). Here, a Bonferroni post-hoc analysis indicated that only *the SVM* and *the regularized OLE* significantly outperformed the other classifiers (p<0.001) both when decoding the position of the first visual flow (p<0.05) and the instructed position of attention (p<0.001, accompanied here by *the reservoir* and *the ANN OLE*). This difference between the absolute performance and the present relative performance analyses was due to the higher 95% confidence limit of the *Reservoir* and *the ANN OLE* as compared to that of the *SVM* and the *regularized OLE.*

### Who's best? Comparing the readout performance for endogenously driven vs. exogenously driven neuronal information

The next question we sought to answer is whether the performance of classifiers on exogenous information is predictive of their performance on endogenous information. We thus compared the performance of the different classifiers at decoding, from the whole FEF population, either the spatial position at which the first stream was presented (exogenous, figures 4a–b, light gray bars) or the position at which attention was instructed by the cue (endogenous, figures 4a–b, dark gray bars). All decoders (*SVM, Reservoir, regularized OLE, Bayesian, ANN OLE or ANN NLE*) provided both a better absolute and relative readout of the exogenous variable as compared to the endogenous variable (2-way ANOVA, Variable x Classifier, Variable main factor, p<0.001, figures 4a–b). Specifically, the average absolute decoding performance of first stream position over all decoders (mean  = 93.0%, s.e. = 5.2%) was 16 percent higher than the average absolute decoding performance of the instructed position of attention (mean  = 77.0%, s.e. = 1.6%). Likewise, the average relative decoding performance of first stream position over all decoders (mean  = 33.8%, s.e = 1.8%) was also 16 percent higher than the average relative decoding performance of the instructed position of attention (mean  = 17.7%, s.e = 1.8%).

Most of FEF neurons encode visual information, while only a small proportion of cells encode the instructed position of attention (16%, [25]). This could account for the higher performance obtained at decoding the exogenous information as compared to the endogenous information. Alternatively, this difference could be due to a noisier encoding of endogenous variables by cortical neurons as compared to how exogenous information is encoded (or more broadly speaking, to different cortical encoding schemes as a function of the variable being considered). In order to address this issue, we performed two additional analyses: 1) we evaluated the decoders' performance at reading out the instructed position of attention from a subset of FEF cells characterized by a statistically significant cue-instruction related response (n = 21), and 2) we evaluated the *SVM's* performance at decoding the first visual stream position from a random selection of 21 visual cells. We then compared the performance a) between the two conditions (population size hypothesis), and b) between the first condition and when using the whole neuronal population (population selectivity hypothesis).

#### Population size hypothesis

**Figure 5:**
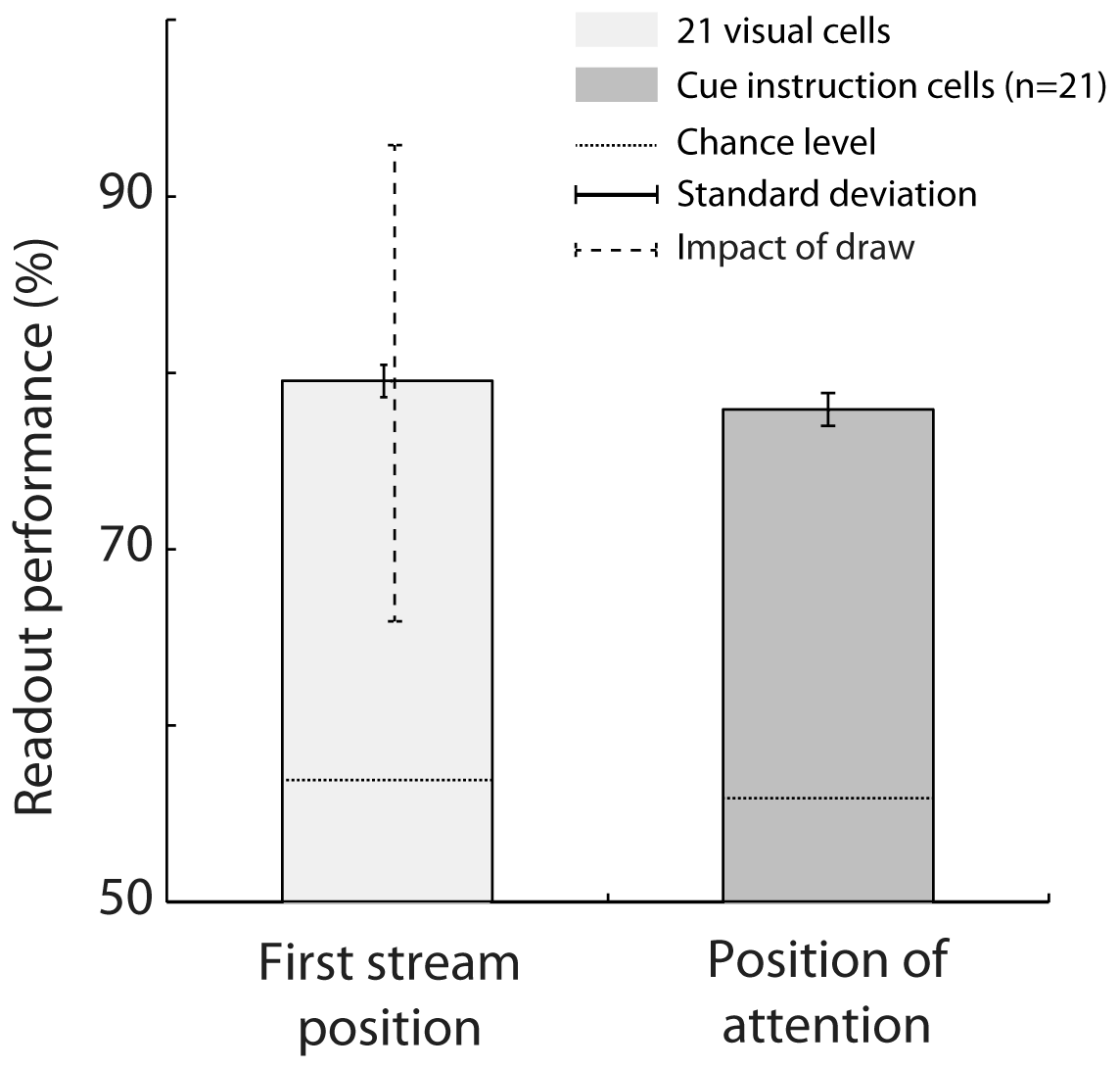
Comparison of decoding flow onset (light gray) with 21 visual cells versus decoding spatial position of attention (dark gray) with the 21 cells with significant individual attention-related responses. The mean read-out performance across 20 runs is showed with standard deviation around this mean. The dotted line corresponds the maximum- and minimum performance across 20 draws of 21 visual cells out of 111. The SVM classifier was used. The mean readout performance and the associated standard error around this mean are calculated over 20 decoding runs. Chance level is defined using a random permutation procedure (p<0.05).

In order to test whether population size fully accounts for the difference in performance between the readout of first visual stream position and the readout of the instructed position of attention, we proceeded as follows. We identified, within the whole FEF population, the visually responsive neurons (n = 111, significant visual modulation within 150 ms from first stream onset for 30 ms out of 25 ms, t-test, p<0.05). We randomly selected 21 visual neurons from this pool of 111 visual neurons and we calculated the average performance of the *SVM* at reading out the first visual stream position from this small population over 20 successive decoding runs. This procedure was repeated 20 times so as to have an estimate of the influence of the cell sampling on the readout performance. Such a procedure yields an absolute average readout performance of 79.5% (s.e. = 0.2%; relative mean performance =  22.6%, s.e. = 0.19% figure 5, light gray bar). Both the absolute and relative average readout performance of attention position from the cue-instruction selective FEF cells fell within the range of readout performance of first flow position from a random small FEF population(absolute performance: p = 0.09, mean = 77.9%, s.e. = 0.90%; relative performance: p = 0.53, mean  = 22.0%, s.e = 0.92%, figure 5, dark gray bar). The smallest readout performance of first visual stream position obtained from the different random samples of FEF visual cells was 65.9% while the highest performance was 92.9% (figure 5, dotted line on light gray bar). This demonstrates that for small populations, performance is highly dependent on the population sample. This applies to the decoding of first visual stream position and most probably also to the decoding of the instructed attention location.

#### Population selectivity hypothesis

The readout performance at decoding the instructed position of attention was estimated from a subset of cells, individually encoding the final cue instruction (21 cells). As for decoding the instructed position of attention from the entire FEF population, all decoders did not perform equally well (one-way ANOVA with repeated measures, decoder main factor, p<0.001, figures 4a–b, medium gray bars). A Bonferroni post-hoc analysis indicated that *the SVM, the regularized OLE, the reservoir*, *the ANN OLE* and *the Bayesian* classifiers outperformed *the ANN NLE* (absolute performance: p<0.001, figure 4a; relative performance: p<0.05, figure 4b). In addition, decoding the instructed position of attention from the whole FEF population or from a selected subset of cells did not affect the readout performance of all decoders in the same way (two-way ANOVA, significant interaction between the two populations and decoder main factors, p<0.001). A Bonferroni post-hoc analysis revealed that this population effect is specific to the *Bayesian decoder* both for the absolute and the relative performances (p<0.001, figure 4a–b), the absolute readout performance of this classifier being 9.0% higher when the decoding is performed on the selected subset of cells than when it is performed on the entire the FEF population.

### Trade-off between population size and population response sampling

Two parameters are expected to drastically influence readout performance: population size (i.e. the number of cells which are simultaneously being recorded from) and population response sampling (i.e. the number of trials on which the training is performed). In the following, we consider sequentially the impact of each parameter in conjunction and then independently so as to gain a better understanding of the contribution of each of these two parameters onto decoding performance. The *ANN NLE* was excluded from all further analysis due to its extremely time costly computations (∼6 hours per data seed/run) combined with a relatively poor readout performance (Regularized OLE/Bayesian: less than 1 second per data seed per run; ANN OLE: less than 2 seconds per data seed per run; SVM/Reservoir: less than 3 seconds per data seed per run; note that these time estimates are both dependent on the type of processor being used and on the optimization of the computation scripts).

**Figure 6:**
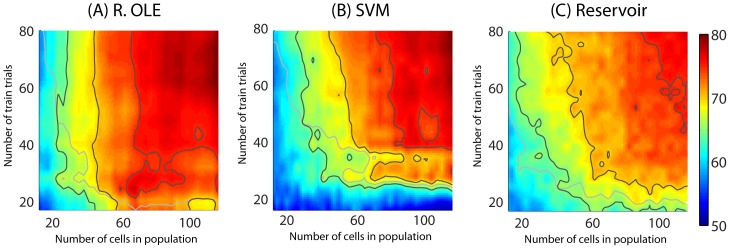
Decoding of spatial attention from the whole FEF population activities as a combined function of number of trials and cells with (A) Regularized OLE, (B) SVM and (C) Reservoir decoders. The black contour lines correspond, from yellow to dark red regions, to 65, 70 and 75% of readout performance. The gray contour lines corresponds to chance level as calculated, at each point, by a random permutation test (p<0.05). Smoothing with Gaussian kernel of 7. The readout performance is an average readout performance on 10 decoding runs. The maximum possible number of training trials is 84 trials. The y-axes are truncated at 80 trials.

Population size and trial number trade-off. In order to further explore the trade-off between trial number and population size when decoding the instructed position of attention from randomly selected FEF cells, we performed an additional analysis in which we co-vary both parameters simultaneously. This analysis is performed on the best performing classifiers, namely, the regularized OLE (figure 6a), the SVM (figure 6b) and the reservoir (figure 6c). On all plots, we indicate both the 65%, 70% and 75% performance iso-contours (figure 6, black contours) and the 95% confidence limits for significant readout (figure 6, gray contours). Confirming our previous observations, the regularized OLE achieved the best readout performance at all population sizes and training trial number combinations. In particular, a 75% absolute performance rate was achieved with as few as 60 cells and as little as 40 training trials. The SVM came next, followed by the Reservoir, although the latter appears to outperform the former for small trial numbers and small population size.

#### Population size

**Figure 7:**
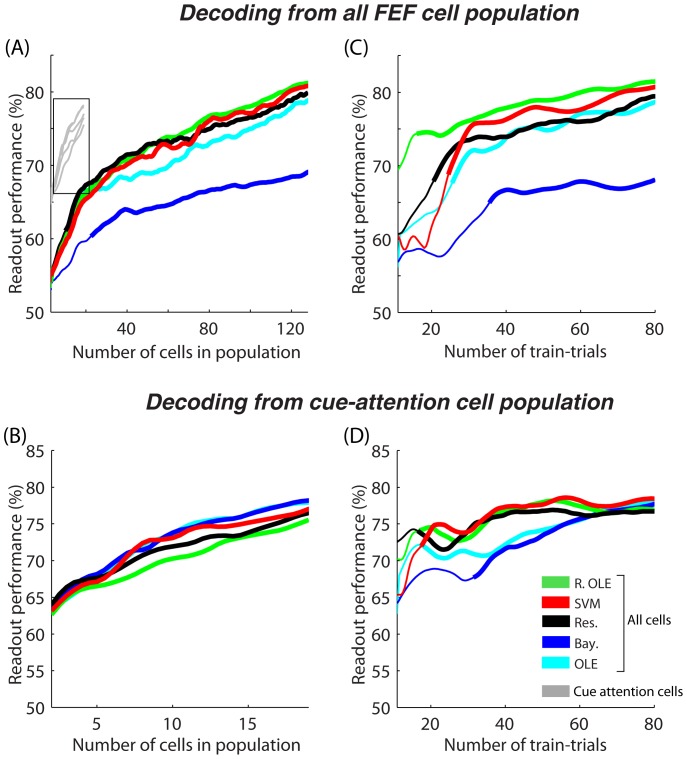
Decoding spatial attention (A–B) as a function of cell population size and (C–D) number of trials available for training. In (A) and (C), decoding is performed on the whole FEF cell population while in (B) and (D), decoding is performed only on the attention-related cells -presented also in gray in (A). The mean readout performance is calculated over 20 decoding runs. Thick lines indicated values that are significantly above chance as calculated using a random permutation test (p<0.05). SVM  =  support vector machine, Res  =  reservoir, Ex. OLE  =  explicit OLE, Bay.  =  Bayesian, NLE  =  ANN non-linear estimator, OLE  =  ANN optimal linear estimator.

The readout performance at decoding the instructed position of attention from the entire FEF population steadily increased as a function of population size for all decoders (Figure 7a). For populations of less than 25 randomly selected FEF neurons, *SVM, Reservoir, regularized OLE* and *ANN OLE* provided equivalent readout success rates, outperforming *the Bayesian* classifier. As the number of neurons in the population increased, *the SVM, the regularized OLE* and *the reservoir* improved their performances similarly whereas *the ANN OLE* improved with a slower rate. The *Bayesian* was trounced by all the others and the impact of increasing the population size onto its readout performance was the lowest.

Absolute readout performances above the upper 95% confidence limit are indicated, in figure 7a, by a thicker line. It is interesting to note that *the SVM* and *the regularized OLE* had an absolute performance significant with as few as 4 random FEF cells. The *reservoir* achieved significant readout performances with 9 cells, whereas *the Bayesian* required 22 cells in the population.

When decoding was performed on the subset of attention selective FEF cells (n = 21, figure 7b), the overall effect of population size on readout performance was equivalent across decoders, except for the fact that the *regularized OLE* improved with a slightly slower rate as the population size increases (Figure 7b). As expected by their high attention-related information content, adding an attention-cell to the population induced an average increase of 0.76% on the readout performance (Figure 7b). This is to be contrasted with the impact of a randomly selected cell onto the overall population performance (0.17% increase in readout performance, figure 7a).

#### Trial number

As for population size, the readout performance at decoding the instructed position of attention from the entire FEF population steadily increased as a function of the number of available trials on which to train the decoders (Figure 7c). However, all decoders did not behave equally in the face of trial number. In particular, the regularized OLE outperformed all other decoders at all values of training trial number. This classifier actually reached significant decoding rates with as few as 10 trials (thick green line, figure 7c). The performance of the Reservoir, SVM and ANN OLE decoders became statistically significant around 20 training trials and stabilized for 30 trials or so (thick lines, figure 7c). While the SVM achieved the best readout performance amongst these three, the Bayesian decoder was outperformed by all the other classifiers at all training trials number and required more than 35 trials to achieve significant readouts.

When the decoding of instructed position of attention was performed on the subpopulation of attention-selective FEF cells, the impact of number of trials was drastically reduced (figure 7d). Indeed, the *regularized OLE*, the *SVM* and the *reservoir* achieved significant readout rates and are close to their maximum decoding performance with as few as 15 cells. The rise to maximum performance was slower for the *ANN OLE* and the *Bayesian* classifiers, and here again, this latter decoder required more data samples to achieve significant readouts (more than 30 trials).

### Training sample balance

**Figure 8:**
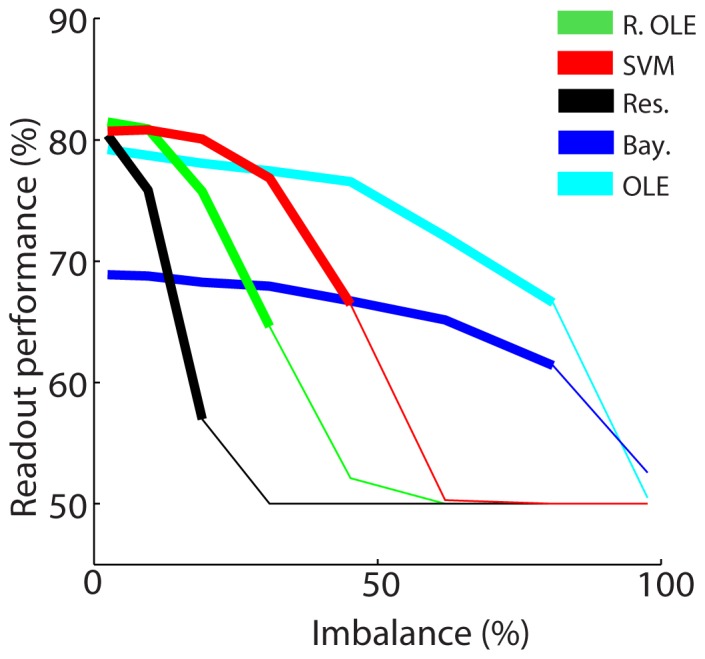
Impact of imbalance in the training set. The y-axis represents the difference between the readout performance of a balanced data set (same number of trials for each condition) and that of an unbalanced data set (more trials in condition 1 than in condition 2). The x-axis represents the degree of imbalance in training trial number between the two conditions. The mean readout performance and the associated standard error around this mean are calculated on 20 decoding runs. Thick lines indicated values that are significantly above chance as calculated using a random permutation test (p<0.05). SVM  =  support vector machine, Res  =  reservoir, R. OLE  =  regularized OLE, Bay.  =  Bayesian, NLE  =  ANN non-linear estimator, OLE  =  ANN optimal linear estimator.

In an online-decoding environment, training is ideally performed on a fixed number of past trials in reference with the testing time-point. The assumption that these fixed trials equally represent condition 1 (here, attention instructed to the left) and condition 2 (attention instructed to the right) might actually be violated, in particular due to a potential bias in the performance of the subject, having a higher performance for one condition over the other. Here, we explored the impact of such an imbalance in the number of training trials for the two states of the variable of interest (figure 8). The overall picture is that this imbalance incurs a drop in average readout performance. This drop in performance increased as the imbalance between the number of trials for the two conditions increased. The rate at which the performance decreased highly depended on the classifier. The *Bayesian* and the *ANN OLE* performed best with a respective performance drop rate of 3% and a 5% for a 50% imbalance in the data set (i.e. when one class has half as many trials as the other class). Furthermore, the *Bayesian* and the *ANN OLE* were the only classifiers for which performance remained above the upper 95% confidence limit at 50% imbalance. In comparison, the *SVM* had an 18% performance drop rate, the *regularized OLE*, a 28% performance drop rate and the *reservoir, a 30%* performance drop rate. There thus appears to be a trade-off between decoding performance in ideal settings and resistance to actual real data biases as considered here.

### Memory

**Figure 9:**
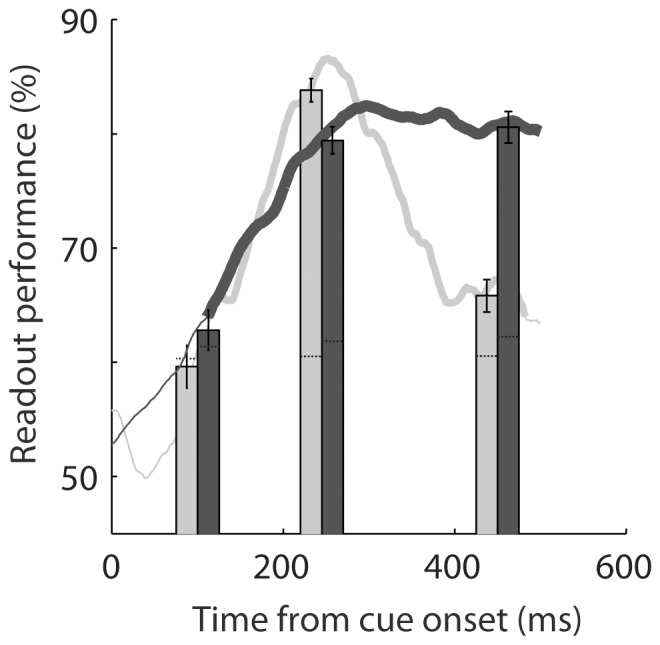
Impact of memory on Reservoir decoding performance on reading out the spatial position of attention. The light gray curve and bars corresponds to a reservoir training on a window of 75(as in all previous figures). The dark gray curve and bars corresponds to a reservoir training a larger time window (from cue onset at 0 ms to 700 ms post-cue). Decoding is performed on all FEF cell population activities. The bars show the mean readout performance and the associated standard error around this mean obtained by testing activities in a time window of 100 ms around the time reference point for training (245 ms after cue onset, N = 20 decoding runs). The curves show the mean readout performance and the associated standard error around this mean for each time point. Thick lines indicated values that are significantly above chance as calculated using a random permutation test (p<0.05).

All previous decoding procedures relied on the estimation of the readout performance from population activities averaged over successive 100 ms windows, irrespectively of the response that was produced by the population at previous time points. However, recent evidence suggests that reverberating activities in local neuronal populations allows to maintain as well as to accumulate information in time [9], [45]. The specific *Reservoir* architecture allows us to directly assess the impact of information maintenance and accumulation over time by simply presenting the network with training data sampled over a longer time interval (70–500 ms after cue onset–figure 9, dark gray curve- versus 212–283 ms–figure 9, light gray curve) while still testing over successive 100 ms intervals (dark and light gray curves respectively, figure 9). In this analysis the classifier is tested on all time points ranging from 70 to 500 ms after cue presentation and each readout performance corresponds to the exact performance for that time point (i.e. in contrast with the previous measures, we do not average the readout performance over a 100 ms window). In this analysis, trials in which the target appeared 150 ms or 300 ms after cue onset have both been excluded to avoid the potential confound between cue and target-related activities. Readout performances above the upper 95% confidence limit are represented with a thick line. As expected, taking into account a longer period of time when training the *reservoir* network resulted in an increased decoding performance throughout the post-cue period, that was sustained at a distance from the cue (400–500 ms post-cue, dark gray curve). Taking into account the temporal structure of the signals however lead to a 5% drop in readout performance at the time of maximum attention-related population activity (245 ms following cue onset). As a result, this decoding approach is only interesting when the ability to track the information over time is more important than achieving maximum decoding performance.

## Discussion

Our results suggest that endogenous information such as the orientation of attention can be decoded from the FEF with the same accuracy as exogenous visual information. In addition, all classifiers did not behave equally in the face of population size and heterogeneity, the available training and testing trials, the subject's behavior and the temporal structure of the variable of interest. In most situations, the regularized optimal linear estimator and the non-linear Support Vector Machine classifiers outperformed the other tested decoders.

### Decoding of endogenous information as compared to exogenous information

Our decoders achieve, on average, a 19% higher performance at decoding exogenous information (here, the position of the first visual stream) from a heterogeneous FEF neuronal population, as compared to endogenous information (here, the position of attention instructed by the cue). These observations are in line with a previous study also showing a higher accuracy at decoding the position of a visual cue (*SVM* classifier, 100% accuracy, [4]) as compared to decoding the position of attention away from cue presentation (*SVM* classifier, 89% accuracy), from a heterogeneous FEF population. In the current study, we further show that this advantage at decoding exogenous over endogenous information is constant across both linear and non-linear classifiers. This could be due to the fact that FEF contains more visual-selective than attention-selective cells (*Cell selectivity hypothesis*). Alternatively, it could be that visual information is encoded in the FEF with a higher reliability than attention-related information (*Response reliability hypothesis*). While we cannot favor one possibility over the other, both are worth considering.

#### Cell selectivity hypothesis

The frontal eye fields are known to have strong, short latency visual responses [24], due to direct as well as indirect anatomical projections from the primary visual cortex V1 [46], [47]. Early studies report that up to 47% of FEF neurons are visually responsive [48] while up to 80% of pre-saccadic FEF neurons are also visually responsive [23]. In the dataset used in the present work, FEF neurons were recorded on the basis of their responsiveness to the key events of the cued-target detection task. Eighty-four percent of these neurons had significant neuronal responses to first visual stream onset (111 visual neurons out of a total of 131 neurons).The frontal eye fields are also known to be at the source of covert attention signals [49], [26]. And indeed, FEF neurons have been shown to encode spatial attention signals. The proportion of such FEF neurons varies from one study to another, most probably due to the specificities of the behavioral task being used. For example, in classical cued-target detection tasks that allow to manipulate spatial attention, the spatial mapping between the cue and the subsequent covert attentional orientation changes. The cue can be a spatial cue, indicating that attention should be held at the location where it is presented. In this case, there is a direct mapping between the location of the cue and the instructed position of attention and about half FEF neurons are shown to represent this latter information (40.8% in [50]; 51.8% in [4]). The cue can be a symbolic cue that requires to be interpreted so that the instructed location of attention can be extracted, for example, a central cue that instructs attention to the right if of a specific type (e.g. red or right pointing arrow), and to the left if of another type (e.g. green or left pointing arrow). In this case, the spatial location of the cue is irrelevant to define the final position of the cue, while its identity is fully informative. Gregoriou et al. [51] report that 44.7% of FEF neurons were modulated by spatial attention in such a task. A more complex situation is the one used in Ibos et al. [25], in which the spatial location and the color of the cue are non-informative if considered separately, but fully informative if combined. This complex transformation most probably accounts for the lower proportion of attentional neurons available in the present dataset (16%, 21 out of 131, [25]). Overall, the proportion of visual and attentional FEF neurons thus appears to vary from one study to another, depending on the specific tasks being used and the associated recording biases.

Focusing on the present dataset (84% of visual cells and 16% of attention-related cells), the better readout performance at decoding the position of the first visual stream from the entire FEF population as compared to the decoding of the instructed position of attention could be due to the fact that more cells contribute to the encoding of this visual event. Constrained by our FEF neuronal sample, we cannot increase the proportion of FEF attention selective cells to match that of visually selective cells. However, we can select amongst the visually-selective cells a random sub-sample of neurons matching the number of attention-selective cells. As described in the cell drop-out analysis, decreasing the size of the neuronal population being decoded from is expected to have a drastic impact on the readout performance. This is indeed what is observed (test performed selectively with the *SVM* classifier, figure 5), though the decoding accuracy highly depends upon the visually-selective cells composing the random sub-sample: in 20 successive draws of a sub-sample of 21 visually-selective cells, performance varied from as low as 65.9% to as high as 92.9%. The readout performance at decoding the instructed position of attention from the attention-selective cells lies within this range. This suggests that the decoding accuracy of visual information and attention information are comparable and that another sample of attention-selective cells could have led to either higher or lower performances than what we describe here. Extrapolating over this observation, it should thus be possible to achieve spatial attention allocation readout performances equal to those obtained for first visual stream onset position, provided more attention-selective cells are included in the neuronal population. This will need to be confirmed experimentally.

#### Response reliability hypothesis

The observed differences in performance at decoding first visual stream position versus the spatial attention allocation could be due to the fact that the encoding of endogenous variables is more susceptible to trial-to-trial variability due to intrinsic factors such as motivation or fatigue. The encoding of a sensory stimulus (as first visual stream onset, here) is expected to be less affected by these intrinsic factors unless its detectability is highly degraded. Supporting this hypothesis, Cohen et al. [52], [53] show that, on a single trial, the degree to which a neuronal V4 population encodes spatial attention varies and is predictive of the overt behavioral performance on that very same trial. In Farbod Kia et al. [29], we demonstrate that, in the present task, part of the error trials arise from a miss-encoding of attention orientation. Here, the run-to-run variability in the decoding accuracy, each run consisting of a different training/testing set of trials, reflects the trial-to-trial variability with which a given variable is encoded by the neuronal population. The decoding accuracy for the spatial position of attention has a higher standard error than the decoding accuracy for first stream position. This could be due to a genuine difference in the trial-to-trial variability with which these two types of information are encoded. It is however worth noting that, though the cue-to-attention mapping required from the monkeys in the present dataset is complex, the *SVM* achieves a readout performance of 81.2% at decoding the spatial allocation of attention from the entire FEF population. This performance is relatively close to that achieved with the same classifier at decoding the same information during a simpler task involving a direct spatial mapping between cue position and attention allocation (89%, [4]) from an FEF population composed of a higher proportion of attention-selective cells (51.8%, in [4], vs. 16% in the present study). This indicates that the proportion of attention-selective cells in the neuronal population is not the only determinant of performance and response variability needs to be considered. Information redundancy across attention-selective cells should also be taken into account. In the current study, as well as in the Armstrong et al. study, single-neuron recordings were achieved in independent sessions. Decoding from simultaneously recorded neuronal population activities in a single animal is expected to uniformly improve readout performance for all decoders due to a decrease in overall (inter-subject and inter-session) data variability. However, the response of simultaneously recorded neurons also shows an important degree of correlation [54]. The impact of these correlations on the total information conveyed by such a neuronal population is controversial [55], [56], [57], [58], preventing a direct estimate of their net effect on the decoding accuracy reported here. This needs to be borne in mind when considering the present study.

Overall, our study suggests that endogenous information such as covert attention orientation can be decoded from an appropriate neuronal population with similar accuracy as exogenous information such as the position of visual stimulus. Interestingly, and in line with our present work, Gunduz et al. [59] show that the spatial position of attention can also be decoded from larger distributed neuronal populations in humans, as recorded from a parieto-frontal ECoG matrix, with a performance of up to 48% (chance  = 33.3%, decoding being performed on the whole band signal spectrum). This decoding accuracy is to be compared to the performance at decoding attentional engagement (84.5%, chance  = 50%) and motor engagement (92.5%, chance  = 50%). Rotermund et al. [60] decode the spatial position of attention, in non-human primates, with a maximum accuracy ranging between 93% (left/right hemisphere spatial attention allocation) and 99% (spatial attention allocation to two close by positions within the same hemisphere), from a large distributed neuronal population, as recorded from an epidural ECoG matrix placed over the striate and extra-striate visual cortex. Altogether, these different studies and ours strongly support the idea that endogenous cognitive information content can be decoded from population neuronal activities.

### The optimal classifiers

A general observation from our study is that the *SVM*, the *Regularized OLE,* the *Reservoir* and *the ANN OLE* unambiguously outperform *the Bayesian* and *the ANN NLE.* A link is often made between reservoir computing and kernel machines [61], [62], in particular because both techniques map the input data into a higher-dimensional feature space. In the case of the *Reservoir*, this mapping is performed explicitly by the reservoir neurons whereas the *SVM* uses the so-called “kernel-trick” to avoid this costly explicit computation. The *Regularized OLE* and *the ANN OLE* differ significantly from these two classifiers because they only use a simple hyperplane to separate the input data (i.e. they can only classify linearly separable data). Even though these four classifiers outperform the other classifiers, there are several other factors that also need to be considered.

#### Temporal structure in decoded feature

A major difference of reservoir computing is that it can depend on the recent history of the input. Such a *Reservoir* allows to process information that is explicitly coded in time. In contrast, the state of the other classifiers only depends on the current input [63]. As a result, using the *Reservoir* classifier is a better choice when decoding variables with a specific temporal organization as is often the case with spatial attention that moves around in time. Indeed, in such a behavioral context as the one described here, attention needs to be sustained in time from cue interpretation up to target detection. When this temporal aspect is taken into account by training the *Reservoir* on single trial population responses sampled over a longer post-cue interval (70–500 ms rather than 207–283 ms), the decoding accuracy for the spatial attention orientation is remarkably maintained over time. However, if the objective is to achieve highest decoding performance, than simpler decoding schemes appear to be more appropriate than *Reservoir* decoding.

#### Decoding speed-accuracy trade-off

Although the *SVM*, *Regularized OLE, Reservoir* and *ANN OLE* perform equally well in an optimal situation, it is important to note that the *regularized OLE* appears to be more resilient to a limited number of trials. Moreover, when both the number of available trials and cells in the population are limited, *the regularized OLE* outperforms *the SVM, reservoir* and *ANN OLE.* Last, when decoding speed becomes critical, the *Regularized OLE* approach is the fastest.

#### Information within the neuronal population

Here, we describe that the *SVM*, *the Regularized OLE, the Reservoir* and *the ANN OLE c*lassifiers outperform the other classifiers when decoding a given feature from a heterogeneous population containing both feature-selective neurons and non-selective neurons. This represents an advantage in an online decoding perspective, as it indicates that optimal readout performance can be achieved without a prior selection of the neuronal population contributing most to the feature of interest. If, for specific purposes, this selection becomes crucial, it can be performed statistically, using for example a single value decomposition approach (SVD, as in [12]).

#### The subject's behavior

Another critical aspect to take into consideration is the behavior of the subject which can also influence the choice of classifier. Indeed, if the subject presents a difficulty to perform the task correctly and is for example biased for one state of the feature of interest, then this produces an imbalance in the training set that can lead to a decrease in the performance. All classifiers do not behave equally in the face of this imbalance. The *Bayesian* and *the ANN OLE* decoders appear to be quite resilient to this factor, while the *SVM*, the *Regularized OLE* and the *Reservoir* are strongly affected by an imbalance beyond 10 to 40%. While imbalance in the training data sample affects the decoding performance of the *SVM* and of the *Regularized OLE*, we have shown that these two classifiers are quite resilient to a drop in trial number. As a result, they can still be considered as optimal in the case of biased behavior, provided the training is performed on a balanced subset of the data.

#### Number of feature states to be decoded

Support vector machines were originally designed for binary classification [41] and there is a lot of ongoing research on how to effectively extend them to multiclass decoding. Up to now several methods have been proposed where a multiclass *SVM* is constructed by using many binary *SVM* classifiers. Generally, this results in a more computationally expensive classifier [64]. The *Regularized OLE*, the *ANN OLE* and the *ANN NLE* are by essence continuous classifiers (as their output can take any value in a one-dimensional, two-dimensional or n-dimensional space) but they can also be extended to multiclass decoding by constructing several binary classifiers. The *Reservoir* can easily be implemented in a multiclass decoding problem thanks to an architecture that has the same number of output neurons as the number of classes. Each output neuron then represents one class, and the output neuron with the highest activation is chosen as best guess on a given trial. It can also be extended to a continuous n-dimensional decoder, reading out for example the position of a given variable in space, thanks to two output cells representing respectively the x- and y-coordinates. The naïve *Bayesian* classifier also naturally extends to multiclass decoding since it calculates the probability of each class given a certain response and then chooses the class with the highest probability. It can be extended to a continuous n-dimensional feature space within the Gaussian process regression framework [65].
